# Real-time urinary electrolyte monitoring after furosemide administration in surgical ICU patients with normal renal function

**DOI:** 10.1186/s13613-016-0168-y

**Published:** 2016-07-22

**Authors:** Luca Zazzeron, Davide Ottolina, Eleonora Scotti, Michele Ferrari, Paola Bruzzone, Silvio Sibilla, Cristina Marenghi, Luciano Gattinoni, Pietro Caironi

**Affiliations:** Dipartimento di Fisiopatologia Medico-Chirurgica e dei Trapianti, Fondazione IRCCS Ca’ Granda – Ospedale Maggiore Policlinico, Università degli Studi di Milano, Via F. Sforza 35, 20122 Milan, Italy; Dipartimento di Anestesia, Rianimazione ed Emergenza Urgenza, Fondazione IRCCS Ca’ Granda – Ospedale Maggiore Policlinico, Milan, Italy; Department of Anesthesiology and Intensive Care Medicine, Georg-August-University Göttingen, Göttingen, Germany

**Keywords:** Furosemide, Loop-diuretics, Urinary sodium, Urinary chloride, Metabolic alkalosis

## Abstract

**Background:**

Although the loop-diuretic furosemide is widely employed in critically ill patients with known long-term effects on plasma electrolytes, accurate data describing its acute effects on renal electrolyte handling and the generation of plasma electrolyte alterations are lacking. We hypothesized that the long-term effects of furosemide on plasma electrolytes and acid–base depend on its immediate effects on electrolyte excretion rate and patient clinical baseline characteristics. By monitoring urinary electrolytes quasi-continuously, we aimed to verify this hypothesis in a cohort of surgical ICU patients with normal renal function.

**Methods:**

We retrospectively enrolled 39 consecutive patients admitted to a postoperative ICU after major surgery, and receiving single low-dose intravenous administration of furosemide. Urinary output, pH, sodium [Na^+^], potassium [K^+^], chloride [Cl^−^] and ammonium [NH_4_^+^] concentrations were measured every 10 min for three to 8 h. Urinary anion gap (AG), electrolyte excretion rate, fractional excretion (Fe) and time constant of urinary [Na^+^] variation (τNa^+^) were calculated.

**Results:**

Ten minutes after furosemide administration (12 ± 5 mg), urinary [Na^+^] and [Cl^−^], and their excretion rates, increased to similar levels (*P* < 0.001). After the first hour, urinary [Cl^−^] decreased less rapidly than [Na^+^], leading to a reduction in urinary AG and pH and an increment in urinary [NH_4_^+^] (*P* < 0.001). Median urinary [Cl^−^] over the first 3-h period was higher than baseline urinary and plasmatic [Cl^−^] (*P* < 0.001). During the first 2 h, difference between FeCl^−^ and FeNa^+^ increased (*P* < 0.05). Baseline higher values of central venous pressure and FeNa^+^ were associated with greater increases in FeNa^+^ after furosemide (*P* = 0.03 and *P* = 0.007), whereas higher values of mean arterial and central venous pressures were associated with a longer τNa^+^ (*P* < 0.05). In patients receiving multiple administrations (*n* = 11), arterial pH, base excess and strong ion difference increased, due to a decrease in plasmatic [Cl^−^].

**Conclusions:**

Low-dose furosemide administration immediately modifies urinary electrolyte excretion rates, likely in relation to the ongoing proximal tubular activity, unveiled by its inhibitory action on Henle’s loop. Such effects, when cumulative, found the bases for the long-term alterations observed. Real-time urinary electrolyte monitoring may help in tailoring patient diuretic and hemodynamic therapies.

**Electronic supplementary material:**

The online version of this article (doi:10.1186/s13613-016-0168-y) contains supplementary material, which is available to authorized users.

## Background

The loop-diuretic furosemide is one of the most frequent pharmacological treatments employed in intensive care unit (ICU) [1, 2]. Although generally accepted indications include extravascular fluid accumulation and reduced urinary output [3], in the last decade much attention has been paid on the role of furosemide in the prevention, treatment [4], and early detection [5] of acute kidney injury (AKI). Nonetheless, data supporting these indications are still controversial, and general recommendations regarding furosemide administration are not yet clearly standardized [6, 7].

The most frequent method of administration in ICU is the intravenous bolus [1, 8]. The rationale for such administration relies on its mechanism of action, directly inhibiting the sodium–potassium-2 chloride (Na^+^/K^+^/2Cl^−^) cotransport of the think ascending limb of Henle’s loop (NKCC2) [9, 10]. Consequently to such inhibition, in patients with normal renal function, furosemide leads to the excretion of up to 20–25 % of the filtered Na^+^, and, in parallel, to an alteration of urinary excretion of other main electrolytes [6]. Moreover, its administration is often associated with a variable effect on urinary output and fractional excretion of Na^+^ [2], and with alterations in plasma electrolytes concentrations and acid–base equilibrium [11, 12], often leading to metabolic alkalosis.

Despite its extensive use, accurate data describing the physiological effects of furosemide in patients admitted to intensive care unit (ICU) are still lacking [7]. Except for few reports describing its hemodynamics and neurohumoral effects [13, 14], no studies have accurately investigated heretofore the acute and immediate effects of furosemide on renal electrolytes handling, which may affect both the renal response to its administration and the associated alterations in acid–base equilibrium.

In preliminary studies, we have recently employed a urinary analyzer (K.IN.G.^®^, Kidney Instant Monitoring^®^), allowing quasi-continuous measurement of urinary pH and urinary concentrations of Na^+^, K^+^, Cl^−^ and ammonium (NH_4_^+^) [15]. Therefore, by retrospectively analyzing the minute-by-minute urinary electrolyte profile, we aimed first to elucidate, in a cohort of ICU patients with normal renal function, the acute renal response to a low-dose intravenous bolus of furosemide and its correlation with patient clinical characteristics. Second, we aimed to investigate the relationship between urinary electrolyte excretion and the development of metabolic alkalosis, as the most frequently observed alteration of acid-base associated with loop-diuretic administration.

## Methods

### Study population

We retrospectively analyzed patients admitted between November 2009 and June 2010 to the postoperative ICU of our Institution after major surgery and connected to the urinary analyzer K.IN.G.^®^ (Kardia s.r.l., Milan, Italy). As previously reported [15], we preliminary investigated the applicability of the analyzer K.IN.G.^®^ in a cohort of 200 ICU patients. Inclusion criteria of the current study were intravenous administration of furosemide during ICU stay, as decided by the attending physician for clinical purposes, the connection of the patient to the urinary analyzer K.IN.G.^®^ and a period of at least 3 h of urinary monitoring after the administration. We excluded patients with less than 18 years of age, chronic or acute renal failure, admitted after liver transplantation or low-genitourinary tract surgery, or receiving intra-operatively diuretics. The study was compliant with the 1975 Declaration of Helsinki and was approved by the local Institutional Review Board (study #1961, on 09/07/2013, Ethical Committee of Fondazione IRCCS Ca’ Granda—Ospedale Maggiore Policlinico, Milan, Italy), who waived patient consent based upon the observational nature of the study.

### Study design

We divided the study population into three different groups based upon the duration of urinary monitoring. The first group included all patients enrolled (*single*-*dosegroup*), in which observation period lasted at least 3 h. The second group included patients in whom observation period lasted up to 8 h (*long*-*termgroup*). The third group included patients who received two or more doses of furosemide during urinary monitoring (*multiple*-*dosegroup*).

### Data collection

We collected data regarding patient demographic, anamnestic, baseline clinical characteristics and dosage of furosemide administered. Moreover, we retrieved data regarding hemodynamics, respiratory and renal functionality, arterial blood gas and laboratory analyses just before the administration of furosemide. For patients included in the *multiple*-*dosegroup*, plasmatic electrolyte concentrations and parameters of acid–base equilibrium were obtained before the first and after the last administration. Alterations of acid base equilibrium in patients receiving multiple furosemide administrations were evaluated by using the Stewart approach to acid–base [18] identifying correlations between electrolyte concentrations and acid–base balance variations. The datasets supporting the conclusions of this article are included within the article (and its additional files—see Additional files 1, 2 and 3).

### Urinary analyzer K.IN.G. ^®^

All the patients included in the study were connected, through their urinary catheter, to the urinary analyzer K.IN.G.^®^ before furosemide administration. This analyzer provides quasi-continuous measurements (every 10 min) of urinary pH, concentrations of Na^+^, K^+^, Cl^−^ and NH_4_^+^, as well as urinary output [15]. The measuring principle of the analyzer relies on the potentiometric method, by means of ion-sensible sensors. For this reason, analyses were obtained without any dilution process.

### Calculations and definitions

We calculated urinary excretion rate of electrolytes from urinary output and electrolyte concentrations measured by the urinary analyzer. Glomerular filtration rate (GFR) was estimated with the standard Cockcroft-Gault formula [16]. Based upon their plasmatic concentrations, and their urinary excretion rate, we calculated the fractional excretion of Na^+^ (FeNa^+^), Cl^−^ (FeCl^−^), and K^+^ (FeK^+^) during study period, as follows:

$$ {\text{FeE }} = {\text{ E}}_{\text{U}} / \, \left( {{\text{GFR}}/1000 \, \times \, \left[ {\text{E}} \right]_{\text{PL}} } \right) $$

where FeE denotes the fractional excretion of the electrolyte of interest, E_U_ its urinary excretion rate, and [E]_PL_ its plasmatic concentration.

Urinary anion gap (AG) was defined as the difference between urinary concentrations of all measured cations (i.e., [Na^+^] and [K^+^]) and anions (i.e., [Cl^−^]) [17], whereas plasmatic strong ion difference (SID) as the difference between plasmatic concentration of all measured cations (i.e., [Na^+^] and [K^+^]) and anions (i.e., [Cl^−^] and lactate) [18]. Time constant of urinary [Na^+^] variation ($\tau {\text{Na}}^{ + }_{\text{U}}$) was mathematically defined as the time required observing a decrease in urinary [Na^+^] down to approximately 63 % of its initial increase after furosemide administration.

### Statistical analysis

Comparison of baseline parameters was performed by Student’s *t* test, the Mann–Whitney rank sum test, the Chi-square test or Fisher’s exact test, as appropriate. Variations in urinary pH and electrolyte concentrations over time were analyzed with one-way or two-way ANOVA for repeated measurements, as appropriate. Comparison between linear regressions was performed by employing the test for equal intercept. To investigate the relationship between baseline clinical characteristics and renal response to furosemide administration, study population was divided according to median values of hemodynamic and renal functional parameters, as well as according to the median value of $\tau {\text{Na}}^{ + }_{\text{U}}$. Data are expressed as mean ± SD, or median (25th–75th percentile), as appropriate. Statistical significance was defined as *P* < 0.05. Analysis was performed by using Sigma Plot 12.5 (Systat Software) and SAS 9.2 computer software (SAS Institute).

## Results

### Study population

We enrolled in the study 39 consecutive patients (*single*-*dosegroup*). In 24 patients, observation was prolonged up to 8 h (*long*-*term* g*roup*), whereas in 11 patients study period included also subsequent administrations (*multiple*-*dosegroup*, for a total period of 22 ± 13 h) (Table 1). The dosage of furosemide administered as a single intravenous bolus averaged 12 ± 5 mg. All patients were spontaneously breathing during the entire study period.

**Table 1:** Characteristics of the patients at baseline

Characteristics	Single-dose group (*n* = 39)	Long-term group (*n* = 24)	Multiple-dose group (*n* = 11)
Age (years)	69 ± 11	67 ± 13	69 ± 16
Sex female—no. (%)	15 (38)	7 (29)	4 (36)
BMI (kg/m^2^)	26 ± 5	26 ± 5	25 ± 3
Causes of admission—no. (%)			
Gastrointestinal surgery	12 (31)	4 (17)	5 (46)
Liver surgery	6 (15)	5 (21)	2 (18)
Thoracic surgery	16 (41)	12 (50)	2 (18)
Others	5 (13)	3 (12)	2 (18)
Diuretics	5 (13)	2 (8)	2 (18)
Beta-blockers	6 (15)	2 (8)	2 (18)
RAAS inhibitors	2 (5)	1 (4)	0 (0)
Length of study period (hours)	3	8	22 ± 13
Furosemide dose (mg)	12 ± 5	12 ± 5	38 ± 17
CVP (mmHg)^a^	8.4 ± 3.2	7.7 ± 3.1	10.3 ± 3.1
Diuresis (ml/kg/h)	0.6 ± 0.3	0.8 ± 0.5	0.9 ± 0.3
Creatinine clearance (ml/min)^a^	83 ± 35	73 ± 35	73 ± 44
Arterial pHa^a^	7.42 ± 0.04	7.43 ± 0.03	7.42 ± 0.05
Arterial PCO_2_ (mmHg)^a^	39.9 ± 3.9	39.6 ± 3.6	40.5 ± 4.5
Na^+^ (mEq/L)	138 ± 3	138 ± 3	138 ± 3.0
K^+^ (mEq/L)	4.1 ± 0.4	4.2 ± 0.4	3.8 ± 0.4
Cl^−^ (mEq/L)	107.0 ± 3.6	107 ± 3	109 ± 4.0
Lactate (mEq/L)^a^	1.1 ± 0.5	1.1 ± 0.5	1.2 ± 0.7
SID (mEq/L)^a^	34.1 ± 2.6	33.3 ± 2.1	32.0 ± 1.9
Arterial BE (mmol/L)^a^	1.6 ± 2.6	2.0 ± 2.9	1.8 ± 3.1

Data are reported as mean ± standard deviation, or numbers (%), as appropriate

*BMI* body mass index, *RAAS* renin–angiotensin–aldosterone system, *CVP* central venous pressure, *PCO*_*2*_ partial pressure of carbon dioxide, *Na* ^*+*^ sodium, *K* ^*+*^ potassium, *Cl* ^*−*^ chloride, *SID* strong ion difference, *BE* base excess

^a^Data on CVP were available for 37 patients, whereas data on creatinine clearance, arterial pH, arterial CO_2_, lactate, SID and arterial BE were available for 34 patients

### Urinary pH and electrolytes after furosemide administration as single bolus

**Fig. 1:**
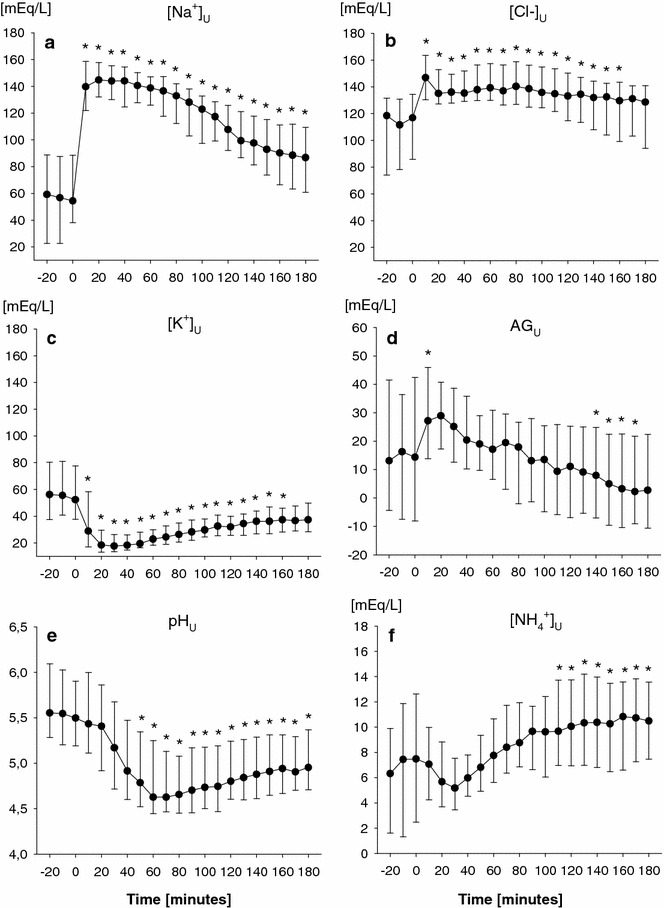
Urinary sodium (Na^+^, **a**), chloride (Cl^–^, **b**), potassium (K^+^, **c**) concentrations, anion gap (AG, **d**), pH (**e**) and ammonium (NH_4_^+^, **f**) concentration every 10 min before and after the administration of an intravenous bolus of furosemide in the overall study population (*single*-*dose* *group*, group *n* = 39). Data are expressed as median and interquartile range. Time 0 (baseline) denotes the time of furosemide administration. One-way ANOVA for repeated measurements was performed (*P* < 0.001 for time effect; *P < 0.05 vs. baseline, time 0)

After furosemide administration, urinary output markedly increased, commencing 10 min after the administration (*P* < 0.05) until the first 100 min (Additional file 4: Figure S1). In parallel, urinary [Na^+^] and [Cl^−^] rapidly increased from 54 (38–88) to 140 (122–159) mEq/l and from 117 (87–134) to 147 (132–163) mEq/l, respectively (Fig. 1a, b). In contrast, urinary [K^+^] fell from 52 (38–77) to 29 (18–56) mEq/L (Fig. 1c) (*P* < 0.001 vs. baseline, for all). After its initial increase, both urinary [Na^+^] and [Cl^−^] slowly decreased toward baseline values, although urinary [Cl^−^] reduction over time appeared less rapid than that of urinary [Na^+^]. After its prompt increase, urinary AG decreased as compared to baseline values (*P* < 0.001 vs. baseline, Fig. 1d). In parallel, urinary pH decreased over time, starting 50 min after furosemide administration (*P* < 0.001 vs. baseline, Fig. 1e), and urinary [NH_4_^+^], progressively increased toward the end of the observation period (*P* < 0.001 vs. baseline, Fig. 1f). Median urinary [Na^+^] and [Cl^−^] over the entire 3-h period increased from baseline to similar values (*P* = 0.09 for electrolyte comparison, and *P* < 0.001 for comparison with baseline values; Table 2). Of note, median urinary [Cl^−^] after furosemide administration was significantly higher than baseline values of plasmatic [Cl^−^] (135 [127–149] vs. 107 [104–110] mEq/l, *P* < 0.001).

**Table 2:** Urinary electrolyte, pH and anion gap profile at baseline and during 3-h furosemide administration

Variable	Baseline	0–30 min	30–60 min	60–90 min	90–120 min	120–180 min	*P*	Median 3 h
[Na^+^]_U_ (mEq/L)	57 (31–88)	142 (130–158)*	141 (127–151)*	134 (113–143)*	116 (97–129)*	91 (105–136)	<0.001	134 (116–142)
[K^+^]_U_ (mEq/L)	56 (39–79)	22 (16–30)*	19 (17–26)*	26 (21–33)*	32 (25–38)*	36 (29–44)*	<0.001	24 (19–32)
[Cl^−^]_U_ (mEq/L)	118 (84–131)	137 (130–153)*	137 (129–155)*	139 (127–156)*	134 (123–152)*	131 (106–142)	<0.001	135 (127–149)
[NH_4_^+^]_U_ (mEq/L)	7.0 (1.5–11.7)	6.0 (4.2–8.6)	6.7 (4.7–9.3)	8.6 (6.6–11.8)*	9.8 (6.9–14.2)*	10.3 (6.8–13.0)*	<0.001	7.6 (5.8–9.4)
Diuresis (ml/kg/h)	1.0 (0.5–1.3)	5.4 (3.4–7.7)*	4.1 (3.0–5.9)*	2.3 (1.7–3.6)*	1.5 (1.1–2.5)*	1.1 (0.8–1.6)	<0.001	2.7 (2.0–3.5)
Diuresis (ml/h)^a^	53 (34–76)	325 (221–394)*		114 (95–179)*		71 (53–100)	<0.001	528 (413–652)
AG_U (_mEq/L)	17 (−4 to 42)	28 (17–40)*	19 (11–31)	17 (0–27)	11 (−6 to 26)	5 (−7 to 23)*	<0.001	20 (8–29)
${\text{Na}}^{ + }_{\text{U}}$ (µEq/kg/min)	0.8 (0.2–1.5)	13.3 (8.0–19.8)*	9.6 (5.9–13.7)*	5.1 (2.9–7.0)*	2.9 (1.9–5.4)*	1.7 (0.9–3.1)	<0.001	6.6 (4.0–8.4)
${\text{K}}^{ + }_{\text{U}}$ (µEq/kg/min)	0.8 (0.5–1.0)	1.9 (1.3–2.5)*	1.4 (1.0–1.7)*	1.1 (0.8–1.4)*	0.8 (0.6–1.0)	0.7 (0.5–1.0)	<0.001	1.1 (1–1.3)
${\text{Cl}}^{ - }_{\text{U}}$ (µEq/kg/min)	1.6 (0.8–2.5)	12.9 (8.2–18.4)*	10.1 (6.4–13.7)*	5.9 (3.8–7.9)*	3.5 (2.6–5.9)*	2.3 (1.7–3.8)	<0.001	7 (4.2–8.2)
${{\text{NH}}_{4}^{ + }}_{\text{U}}$ (µEq/kg/min)	0.09 (0.01–0.22)	0.45 (0.30–0.94)*	0.45 (0.32–0.62)*	0.35 (0.21–0.50)*	0.25 (0.15–0.39)*	0.20 (0.13–0.29)	<0.001	0.34 (0.21–0.47)
FeNa^+^ (%)^b^	0.4 (0.2–1.1)	8.2 (6.1–12.5)*	6.6 (4.1–10.0)*	3.5 (1.8–6.4)*	1.8 (1.0–4.4)*	0.9 (0.6–2.4)	<0.001	3.9 (2.7–5.7)
FeK^+^ (%)^b^	18 (13–35)	47 (38–65)*	35 (25–43)*	28 (17–35)	22 (14–29)	17 (9–25)	<0.001	29 (21 – 35)
FeCl^−^ (%)^b^	1.4 (0.6–2.3)	10.4 (7.1–16.4)*	8.4 (6.5–12.5)*	4.5 (3.4–9.2)*	2.7 (1.9–5.0)*	1.8 (1.1–3.7)	<0.001	5.5 (4.1–8.1)

Data are reported as median value and interquartile ranges of values recorded during 30-min periods before (baseline) and after the intravenous administration of furosemide in the overall study population (*single*-*dose* *group*, *n* = 39). Median 3 h denotes median values of parameters recorded, as average, during the entire 3-h period

$Na^{ + }_{U}$ urinary sodium, $K^{ + }_{U}$ urinary potassium, $Cl^{-}_{U}$ urinary chloride, ${\text{NH}}_{4}^{ + } {\text{U}}$ urinary ammonium, *AG*_*U*_ urinary anion gap, *Fe* fractional excretion

^a^Data on Diuresis reported as between 0–30 min and 60–90 min refer to the total time included between 0–60 min and 60–120 min, respectively

^b^Data on FeNa^+^, FeK^+^ and FeCl^–^ were available for 34 patients. One-way ANOVA for repeated measurements was performed. * *P* < 0.05 versus baseline

**Fig. 2:**
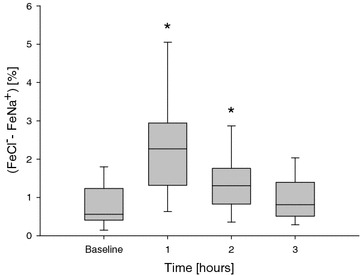
Difference between chloride and sodium fractional excretion at baseline and after furosemide administration. Data are reported as hourly median, interquartile ranges, and 5° and 95° percentile range of the difference between fractional excretion of chloride and fractional excretion of sodium before and after the administration of an intravenous bolus of furosemide in the overall study population (*single*-*dose* *group*, group *n* = 39). Baseline denotes the time of furosemide administration. One-way ANOVA for repeated measurements was performed (*P* < 0.001 for time effect; *P < 0.05 vs. baseline, time 0). *Na* ^*+*^ sodium, *Cl* ^*−*^ chloride

Urinary excretion rates of Na^+^, Cl^−^, K^+^ and NH_4_^+^ increased over time as compared to baseline values (Table 2). These increments appeared significantly different within 30 min after the administration (*P* < 0.05 for all). FeNa^+^ and FeCl^−^ significantly increased within 30 min and remained elevated for 2 h. Hourly values of FeNa^+^, both at baseline and after furosemide administration, were linearly associated with values of FeCl^−^ (*r*^2^ = 0.88 and *r*^2^ = 0.95, respectively, *P* < 0.001 for both), but such relationship significantly shifted upward after the administration (*P* = 0.002 for intercept comparison; Additional file 4: Figure S2). Moreover, the difference between FeCl^−^ and FeNa^+^ significantly increased during the first 2 h after furosemide administration (*P* < 0.05, Fig. 2).

### Short-term variability of furosemide on urinary Na^+^ excretion rate

**Fig. 3:**
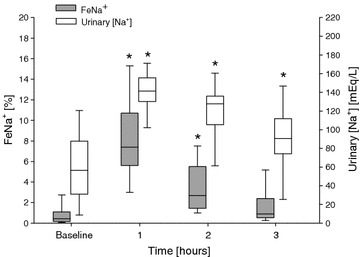
Hourly time course of fractional excretion of sodium (FeNa^+^, *dark bar*) and urinary sodium concentration (Na^+^, *open bar*) before and after the administration of an intravenous bolus of furosemide in the overall study population (*single*-*dose* *group*, group *n* = 39). Data are expressed as median value, interquartile range, and 5° and 95° percentile range. Time 0 (baseline) denotes the time of furosemide administration. One-way ANOVA for repeated measurements was performed (*P* < 0.001 for time effect; *P < 0.001 vs. baseline, time 0)

Although urinary [Na^+^] and Na^+^ excretion rate increased in all patients, these effects appeared widely variable (Fig. 3), ranging from 0.7 to 18.1 % for FeNa^+^ within the first hour, due to the early variation in urinary output, and from 13 to 201 mEq/l for urinary [Na^+^] at the third hour, denoting a variable reduction rate after its first increment. Such variability did not depend on the dose of furosemide administered (*P* = 0.28 and *P* = 0.54, respectively, for FeNa^+^-dose and urinary [Na^+^]-dose interactions).

**Fig. 4:**
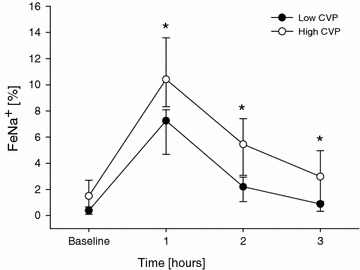
Time course of hourly fractional excretion of sodium before and after intravenous furosemide administration in the overall study population (*single*-*dose* *group*, group *n* = 39), as divided according to the median value of central venous pressure at baseline (8; 6–11). Data are expressed as median value and interquartile ranges. Baseline denotes the time of furosemide administration. Two-way ANOVA for repeated measurements was performed (*P* < 0.001 for time effect; *P* < 0.001 for group effect; *P* = 0.03 for interaction; *P < 0.05 vs. baseline, time 0). *Na* ^*+*^ sodium, *CVP* central venous pressure

When the study population was divided according to baseline hemodynamics and renal functional parameters (Additional file 4: Table S1), patients with higher CVP (>8 mmHg) and FeNa^+^ at baseline (>0.4%) showed a greater increment in FeNa^+^ after furosemide as compared to patients with lower CVP and FeNa^+^, respectively (*P* = 0.03 and *P* = 0.007 for interaction; Fig. 4). When study population was divided according to the median value of $\tau {\text{Na}}^{ + }_{\text{U}}$, patients with a slower decrease in urinary [Na^+^] over time showed a higher baseline MAP and CVP as compared to those with a faster decrease (Additional file 4: Table S2).

### Long-term observation

**Fig. 5:**
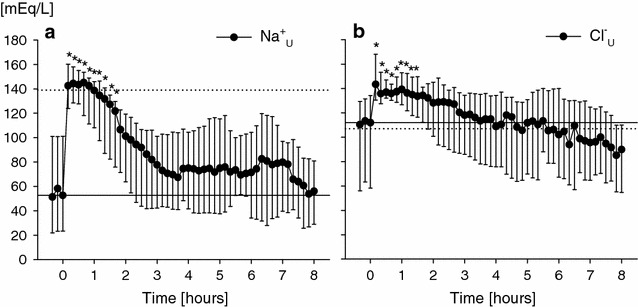
Long-term time course of urinary sodium and chloride concentration after furosemide administration. Urinary sodium (Na^+^, **a**) and chloride (Cl^−^, **b**) concentrations every 10 min before and after the administration of an intravenous bolus of furosemide in the subgroup of patients in which urinary monitoring lasted up to 8 h (*long*-*term* *group*, *n* = 24). Data are expressed as median and interquartile range. Time 0 (baseline) denotes the time of furosemide administration. One-way ANOVA for repeated measurements was performed (*P* < 0.001 for time effect; *P < 0.001 vs. baseline, time 0)

The increase in urinary [Na^+^] and [Cl^−^] observed after furosemide administration lasted mainly 3 h, as median urinary concentrations of both electrolytes achieved baseline values around the third hour (*long*-*termgroup*, *n* = 24, Additional file 4: Table S3 and Fig. 5). A similar time course was observed for urinary [NH_4_^+^]. Urinary excretion rate of all the electrolytes assessed significantly increased as compared to baseline values only within the first 3 h (*P* < 0.05 vs. baseline values), returning toward baseline values in the subsequent hours. Notably, median values of urinary [Cl^−^] over the entire 8-h period were higher than baseline values of plasmatic [Cl^−^] (125 [112–132] vs. 107 [105–109] mEq/l, *P* = 0.02).

In the subgroup of patients receiving multiple administrations (*multiple*-*dosegroup*, *n* = 11), median urinary [Na^+^] and [Cl^−^] during furosemide administration were higher than baseline values (*P* < 0.001 and *P* = 0.03, respectively; Additional file 4: Table S4). Similar results were observed analyzing the respective variations in urinary excretion rate. Moreover, while urinary [NH_4_^+^] did not change during furosemide administration, its excretion rate increased as compared to baseline (*P* = 0.002). Median urinary [Na^+^] and [Cl^−^] during the administration tended to be respectively lower and higher than their plasmatic concentrations at baseline (respectively 128 [118–140] vs. 139 [135–142] mEq/l, *P* = 0.06, for Na^+^; 129 [108–133] vs. 110 [106–11] mEq/l, *P* = 0.06, for Cl^−^). In these patients, multiple administrations of furosemide were associated with an increase in arterial BE (*P* = 0.001), pH (*P* = 0.01) and plasmatic SID (*P* = 0.01), due to a reduction in plasmatic [Cl^−^] (*P* = 0.009, Table 3).

**Table 3:** Plasmatic acid–base equilibrium before and after furosemide administration

	Before	After	*p*
pH	7.43 (7.40–7.46)	7.46 (7.43–7.48)	0.014
PaCO_2_ (mmHg)	40 (38–43)	41 (39–45)	0.36
HCO_3_^−^ (mmol/L)	26.6 (25.3–28.3)	29.0 (28.5–30.6)	0.002
BE (mmol/L)	2.2 (1.5–4.6)	5.5 (4.2–6.8)	0.001
SID (mEq/L)	31 (31–33)	35 (34–36)	0.012
Na^+^ (mEq/L)	139 (135–142)	140 (137–141)	0.36
K^+^ (mEq/L)	3.7 (3.5–4.4)	3.8 (3.5–4.2)	0.92
Cl^−^ (mEq/L)	110 (106–111)	106 (105–109)	0.009
Lactate (mEq/L)	0.9 (0.7–2.2)	1.0 (0.7–1.2)	0.24

Data are reported as median value and interquartile ranges before the first and after the last intravenous administration of furosemide for the subgroup of patients receiving multiple administrations (*multiple*-*dose* *group*, *n* = 11). Total dose of furosemide averaged 38±17 mg, and observation period lasted 22 ± 13 h. Paired *t* test or Wilcoxon signed rank test was performed, as appropriate

*PaCO*_*2*_ arterial partial pressure of carbon dioxide, *HCO*_*3*_^*−*^ bicarbonate concentration, *BE* base excess, *SID* strong ion difference, *Na* ^*+*^ sodium concentration, *K* ^*+*^ potassium concentration, *Cl* ^*−*^ chloride concentration

## Discussion

Our study shows that intravenous administration of furosemide, even at low doses, in patients with relatively normal renal function, induces an immediate subverting of both normal urinary excretion rate and concentration of main electrolytes, which commences very rapidly, and may have subsequent long-term effects as an additive result of sequential administrations.

As a first alteration, in parallel with the rapid increase in urinary output, urinary [Na^+^] constantly increased up to a value close to plasma [Na^+^] (about 142 mEq/L, as average). Notably, such increase did not appear related to baseline urinary [Na^+^] before administration (ranging from 5.0 to 166 mEq/L). Few studies have previously analyzed the time course of urinary [Na^+^] after furosemide administration, being limited to the cumulative natriuresis (over a 24-h period), or the average urinary [Na^+^] over a longer time [2, 19, 20]. In these investigations, urinary [Na^+^] appeared lower than that observed in our study (120–125 mEq/L), but similar, as average, to that measured over the 8-h period, as the possible dilution of the early peaked urinary [Na^+^] with its following reduction. Overall, the urinary [Na^+^] time profile highlights the action of loop-diuretics within the nephron. By inhibiting the cotransport NKCC2 of the Henle’s loop, which generates the hyperosmolar gradient of medullary interstitium [21], furosemide switches off acutely such generation, equilibrating the interstitial osmolality with that of plasma of the peri-tubular capillaries. Therefore, pre-urine arriving to the distal tubules after Henle’s loop inhibition equilibrates with the medullary interstitial space, appearing similar to that of plasma.

Patients with higher values of CVP or FeNa^+^ showed a greater increment in FeNa^+^ after furosemide as compared to those with lower values. Moreover, CVP was higher in patients presenting a slower decrease in urinary [Na^+^] over time (longer $\tau {\text{Na}}^{ + }_{\text{U}}$), as compared to patients with a shorter $\tau {\text{Na}}^{ + }_{\text{U}}$. Expansion of intravascular and right atrial volume have been consistently associated with the release into circulation of both atrial (ANP) and brain natriuretic (BNP) peptides, as a consequence of myocardial stretch [22]. ANP/BNP are thought to promote natriuresis by inhibiting Na^+^ reabsorption in the medullary collecting tubules [23], as well as in the proximal tubule [24, 25]. Although we did not directly measure serum ANP/BNP, we may speculate that the acute resetting of the cotransport NKCC2-dependent hyperosmolality of the Henle’s loop unveils the Na^+^-excretive ANP/BNP-related state characterizing both the proximal and the medullary collecting tubules, especially in patients with greater blood volume expansion.

In parallel with the slow decrease in urinary [Cl^−^] and the urinary [Na^+^]–[Cl^−^] dissociation, we observed a late progressive increase in urinary [NH_4_^+^]. Renal ammonia production predominantly derives from glutamine metabolism in proximal tubular cells [26, 27]. Moreover, pre-urinary NH_4_^+^ competes with K^+^ for the cotransport NKCC2, which is responsible for NH_4_^+^ reabsorption, and NH_3_/NH_4_^+^ recycling within the interstitial medulla [28]. It is conceivable that the late increase in urinary [NH_4_^+^] may be related to an increased aldosterone synthesis and release, enhancing the activity of the luminal H^+^-ATPase of type A intercalated cells [29], thus increasing the acidification of urine and the amount of NH_3_ transformed into NH_4_^+^ and trapped into the lumen [28, 30].

In the subgroup of patients receiving multiple administrations, we observed the development, in about 22 h, of mild metabolic alkalosis. Several studies have investigated loop-diuretics-induced metabolic alkalosis, focusing on its maintenance and recovery [31, 32], whereas few have investigated its generation. Traditionally, the generation of diuretic-induced metabolic alkalosis is considered as related to the contraction of extracellular fluid volume and the consequent increase in HCO_3_^−^ concentration [33]. In contrast, recent studies have clearly pointed out the crucial role of Cl^−^ depletion, as opposed to volume depletion, as the main mechanism maintaining diuretic-induced metabolic alkalosis [32]. According to the Stewart’s approach to acid–base equilibrium, metabolic alkalosis is determined either by a reduced plasma concentration of non-volatile weak acids or by an increased plasma SID [34]. In our study, this alteration appeared associated with an increased plasma SID, due to a reduction in plasma [Cl^−^] [35]. During the 8-h period, median urinary [Cl^−^] significantly increased as compared to baseline, and as compared to baseline plasma [Cl^−^], mainly due to the first 3-h period, in which we observed an early peaked urinary [Cl^−^], and a slower decrement, as compared to urinary [Na^+^] time course. Similarly, in patients receiving multiple doses, median urinary [Cl^−^] tended to be higher than baseline plasma [Cl^−^], likely resulting from the cumulative effect of repetitive administrations, which rapidly increase urinary [Cl^−^].

Which are the mechanisms underlying the higher urinary [Cl^−^] excretion rate, as dissociated from [Na^+^], as the key factor generating hypochloremic metabolic alkalosis? First, the acute “switching-off” of the Henle’s loop unveils the quality of the pre-urine originating at the end of the proximal tubule, which may acutely reach the end of the nephron as unmodified. Along the proximal tubule, luminal [Cl^−^] increases to levels higher than plasma [Cl^−^] because of a different Cl^−^ permeability of the luminal membrane, whereas [Na^+^] remains constant [36]. Second, the slow decrement in urinary [Cl^−^], resulting in an increased urinary [Na^+^]–[Cl^−^] difference, may be due to both a secondary increased activity of aldosterone, promoting a Cl^−^-independent cortical Na^+^ reabsorption, and a reduced activity of the luminal Cl^−^/HCO_3_^−^ exchanger pendrin, mediating Cl^−^ reabsorption and HCO_3_^−^ secretion in type B cortical intercalated cells [37, 38]. We may hypothesize that the long-term effects of furosemide depend on its acute and immediate inhibition on Henle’s loop, unveiling the activity of nephron proximal tubules, which, as an additive effect of sequential administrations, is responsible of the long-term effects observed.

Our study has certain limitations. First, the sample size included is limited, thereby preventing us to fully investigate the possible pathophysiologic mechanisms underlying the urinary electrolyte alterations observed. Second, due to the retrospective and observational nature of the study, we cannot exclude the effects of possible confounding factors, which we were not able to control. Nonetheless, the consistency of the urinary data observed supports the solidity and the biological plausibility of the findings observed. Third, since plasma creatinine is clinically assessed once daily, GFR was estimated, and not directly calculated, therefore partially limiting the accuracy of electrolyte Fe calculations. Finally, no direct data were obtained on the activation of either ANP/BNP or the renin–angiotensin–aldosterone system in parallel with furosemide administration.

Our findings may have also some clinical implications, which warrant further verifications. First, the renal response to furosemide observed in patients with normal renal function provides a pathophysiological rationale for the furosemide stress test to early detect AKI [5], especially when the injury deemed responsible for renal failure is located at the proximal tubular level [39]. Similarly, the acute response observed after furosemide may unveil patient Na^+^-retaining or Na^+^-excretive state independent of Henle’s loop activity, thereby helping in tailoring patient hemodynamic management. The late increase in urinary NH_4_^+^ potentially associated with an aldosterone-induced increase in H^+^-ATPase activity may suggest an increased renal O_2_ consumption following furosemide, in contrast to previous findings [14, 40], which may warn about the use of furosemide in clinical condition at risk of AKI. Finally, our findings highlight the importance of the increased urinary [Cl^−^] excretion rate as the key mechanism generating diuretic-induced metabolic alkalosis. A real-time urinary electrolyte monitoring may represent a potential novel tool to elucidate the specific effects of loop-diuretics in ICU patients, while clarifying its variable efficacy and helping in better tailoring patient hemodynamics therapy.

## Conclusion

In this study, for the first time, we accurately describe in surgical ICU patients with relatively normal renal function the urinary electrolyte excretion profile during the acute phase following the administration of an intravenous bolus of furosemide. Low-dose furosemide administration immediately modifies urinary electrolyte excretion rates, likely in relation to the ongoing proximal tubular activity and patients baseline clinical characteristics, both unveiled by its inhibitory action on Henle’s loop. Such effects, when cumulative, found the bases for the long-term acid–base alterations observed. Real-time urinary electrolyte monitoring may represent an interesting and useful tool for better tailoring patient diuretic and hemodynamic therapies.

## Additional files

10.1186/s13613-016-0168-y Dataset of single-dose group.
10.1186/s13613-016-0168-y Dataset of long-term group.
10.1186/s13613-016-0168-y Dataset of multiple-dose group.
10.1186/s13613-016-0168-y Additional results.

## Abbreviations

AG: anion gap
AKI: acute kidney injury
ANP: atrial natriuretic peptide
BE: base excess
BMI: body mass index
BNP: brain natriuretic peptide
Cl^−^: chloride
CVP: central venous pressure
FeCl^−^: fractional excretion of chloride
FeK^+^: fractional excretion of potassium
FeNa^+^: fractional excretion of sodium
GFR: glomerular filtration rate
H^+^: hydrogen ion
HCO_3_^−^: bicarbonate
ICU: intensive care unit
K^+^: potassium
K.IN.G.^®^: Kidney Instant Monitoring^®^
MAP: mean arterial pressure
Na^+^: sodium
NH_4_^+^: ammonium
NKCC2: sodium–potassium-2 chloride of thick ascending limb of Henle’s loop
PaCO_2_: arterial partial pressure of carbon dioxide
RAAS: renin–angiotensin–aldosterone system
SID: strong ion difference
$\tau {\text{Na}}^{ + }_{\text{U}}$: time constant of urinary sodium concentration variation
