# Supplementary material for: Real-time urinary electrolyte monitoring after furosemide administration in surgical ICU patients with normal renal function
Source: Ann Intensive Care. 2016 Jul 22;6:72. doi: 10.1186/s13613-016-0168-y (PMC4958084; doi:10.1186/s13613-016-0168-y)
Supplement: Supplementary file 4 — 10.1186/s13613-016-0168-y Additional results. [file 13613_2016_168_MOESM4_ESM.docx]

**Supplemental Digital Content**

**Real-time urinary electrolyte monitoring after furosemide administration**

**in critically ill patients with normal renal function**

Luca Zazzeron, M.D., Davide Ottolina, M.D., Eleonora Scotti, M.D., Michele Ferrari, M.D.,

Paola Bruzzone, M.D., Silvio Sibilla, M.D., Cristina Marenghi, M.D.,

Luciano Gattinoni, M.D., F.R.C.P., and Pietro Caironi, M.D.

**Additional Methods**

The study was approved by the local Institutional Review Board (study #1961, on 09/07/2013, Ethical Committee of Fondazione IRCCS Ca’ Granda – Ospedale Maggiore Policlinico, Milan, Italy), who waived patient consent based upon the observational nature of the study.

Time constant of urinary [Na^+^] variation (τNa^+^_U_) was defined as the time required to observe a decrease in urinary [Na^+^] down to approximately 63% of its initial increase after furosemide administration, expressed in minutes. If such threshold was not achieved at the end of the third hour of observation, τNa^+^_U_ was arbitrarily considered equal to 190 minutes.

**Additional Results**

**Supplementary Figure S1 – Effect of an intravenous bolus administration of furosemide on diuresis.**

Time course of urinary output before and after the administration of an intravenous bolus of furosemide in the overall study population (*Single-Dose-Group*, group n = 39). Data are expressed as median value of 10-minutes urinary output (ml/kg/h), interquartile range, and 5° and 95° percentile range. Time 0 (baseline) denotes the time of furosemide administration. One-way ANOVA for repeated measurements was performed (P < 0.001 for time effect; * P < 0.05 vs. baseline, time 0).

**Supplementary Figure S2 – Relationship between hourly sodium and chloride fractional excretion before and after furosemide administration.**

Data represents hourly fractional excretion of sodium in relationship with that of chloride before (solid dots) and after (open dots) intravenous furosemide administration in the overall study population (*Single-Dose-Group*, group n = 39). Both the relationships were analyzed by linear regression analysis (before the administration: FeCl^–^ = 0.595 + (1.253 * FeNa^+^); r^2^=0.88; P<0.001; after the administration: FeCl^–^ = 0.751 + (1.197 * FeNa^+^); r^2^=0.95; P<0.001). *Abbreviations:* Na^+^ = sodium; Cl^–^ = chloride.

**Supplementary Table S1 – Baseline clinical and urinary features and fractional excretion of sodium after an intravenous bolus of furosemide.**

| **Baseline Features** | **Median**  **(25P - 75P)** | **Median**  **(25P - 75P)** | **Patients** | **Dose (mg)** | **FeNa^+^**  **at Baseline**  **(%)** | **FeNa^+^**  **1^st^ hour**  **(%)** | **FeNa^+^**  **2^nd^ hour**  **(%)** | **FNa^+^**  **3^rd^ hour**  **(%)** | **P**  **interaction** | **P**  **group** | **P**  **time** |
| --- | --- | --- | --- | --- | --- | --- | --- | --- | --- | --- | --- |
| **HR (bpm)** | 80  (71-91) | 71 (65-72) | 19 | 10 (10-20) | 0.3 (0.1-0.8) | 7.4 (5.8-8.7) | 2.2 (1.1-4.4) | 0.9 (0.5-1.3) | 0.334 | 0.12 | <0.001 |
|  |  | 91 (87-104) | 20 | 10 (10-10) | 0.6 (0.3-1.8) | 9.2 (5.2-11.7) | 3.4 (2.2-7.3) | 0.9 (0.7-3.4) |  |  |  |
| **MAP (mmHg)** | 85  (77-97) | 77 (72-80) | 19 | 10 (10-20) | 0.3 (0.3-0.9) | 7.3 (5.3-9.0) | 2.4 (1.1-4.4) | 0.8 (0.4-1.6) | 0.185 | 0.281 | <0.001 |
|  |  | 96 (90-101) | 20 | 10 (10-10) | 0.6 (0.3-1.1) | 8.1 (5.8-11.7) | 3.0 (2.1-5.7) | 1.2 (0.7-2.5) |  |  |  |
| **CVP (mmHg)^a^** | 8  (6-11) | 6 (5-7) | 19 | 10 (10-10) | 0.3 (0.1-0.6)*** | 6.2 (4.8-7.4)* | 1.9 (1.1-2.3)*** | 0.7 (0.3-1.0)*** | 0.033 | <0.001 | <0.001 |
|  |  | 11 (10-12) | 18 | 10 (10-20) | 1.0 (0.4-2.6) | 9.5 (8.7-13.5) | 5.6 (3.3-7.7) | 2.3 (0.9-4.8) |  |  |  |
| **GFR (ml/min)^a^** | 66  (57-82) | 57 (43-62) | 17 | 10 (10-20)* | 0.9 (0.2-1.3)* | 8.9 (5.8-13.5) | 4.2 (2.5-6.2)* | 1.7 (0.7-3.0) | 0.358 | 0.047 | <0.001 |
|  |  | 82 (69-97) | 17 | 10 (10-10) | 0.3 (0.1-0.5) | 7.4 (5.8-8.9) | 2.2 (1.1-2.8) | 0.9 (0.6-1.0) |  |  |  |
| **[Na^+^_U_] (mEq/L)** | 57  (31-88) | 31 (10-46) | 19 | 10 (10-20) | 0.2 (0.1-0.4)*** | 5.8 (4.8-9.8) | 2.2 (1.1-3.4)** | 0.7 (0.3-1.0)*** | 0.089 | 0.001 | <0.001 |
|  |  | 88 (83-112) | 20 | 10 (10-10) | 0.9 (0.4-1.8) | 8.9 (7.1-11.7) | 4.4 (2.2-7.3) | 1.3 (0.9-3.4) |  |  |  |
| **V_U_ (ml/kg/h)** | 1  (0.5-1.3) | 0.5 (0.3-0.7) | 19 | 10 (10-10) | 0.3 (0.1-0.5)** | 6.9 (3.8-8.2)* | 2.0 (1.1-2.6)** | 0.7 (0.5-1.0)** | 0.438 | 0.003 | <0.001 |
|  |  | 1.3 (1.1-1.9) | 20 | 10 (10-20) | 0.9 (0.3-1.8) | 9.3 (5.8-13.5) | 4.2 (2.8-7.3) | 1.6 (0.7-3.4) |  |  |  |
| **FeNa^+^_U_ (%)^a^** | 0.4  (0.2-1.1) | 0.2 (0.1-0.3) | 17 | 10 (10-10) | 0.2 (0.1-0.3)*** | 5.8 (4.5-8.9)** | 1.5 (1.1-3.3)*** | 0.6 (0.3-0.9)*** | 0.007 | <0.001 | <0.001 |
|  |  | 1.1 (0.8-1.8) | 17 | 10 (10-10) | 1.1 (0.8-1.8) | 8.9 (7.1-14) | 4.2 (2.5-7.3) | 1.7 (0.9-3.4) |  |  |  |
| **[Na^+^_U_]/[K^+^_U_]^b^** | NA** | 0.4 (0.3-0.6) | 18 | 10 (10-20) | 0.2 (0.1-0.4)*** | 5.8 (4.5-9.2)** | 1.5 (1-3.4.0)*** | 0.5 (0.3-1.0)*** | 0.067 | <0.001 | <0.001 |
|  |  | 2 (1.1-2.7) | 17 | 10 (10-20) | 0.9 (0.4-2.6) | 10.1 (7.4-14) | 5.7 (2.2-7.3) | 2.4 (0.9-4.7) |  |  |  |

Data are reported as median value and interquartile ranges. ^a^ Data on CVP were available for 37 patients, whereas data on GRF and FeNa^+^ for 34 patients, and data on [Na^+^_U_]/[K^+^_U_] on 35 patients. ^b^ Study population was divided according to a [Na^+^_U_]/[K^+^_U_] either greater than 1 or not. Two-way ANOVA for repeated measurements was performed. * P < 0.05, ** P < 0.01, and *** P < 0.001 vs. other group. *Abbreviations:* HR = heart rate; MAP = mean arterial pressure; CVP = central venous pressure; GFR = glomerular filtration rate; [Na^+^_U_] = urinary sodium concentration; V_U_ = urinary output; FeNa^+^_U_ = fractional excretion of sodium; [Na^+^_U_]/[K^+^_U_] = urinary sodium and potassium concentration's ratio.

**Supplementary Table S2 – Urinary properties and baseline clinical variables of the patients by time constant of urinary sodium concentration decrease after furosemide administration.**

|  | **Fast decrease**  **N = 20** | **Slow decrease**  **N = 19** | **p** |
| --- | --- | --- | --- |
| **Dose (mg)** | 10 (10 - 10) | 10 (10 - 20) | 0.10 |
| **Baseline Na^+^_U_ (mEq/L)** | 52 (15 - 99) | 79 (42 - 88) | 0.53 |
| **First Na^+^_U_ (mEq/L)** | 149 (119 - 159) | 134 (126 - 158) | 0.70 |
| **First Na^+^_U_ variation (mEq/L)** | 84 (54 - 108) | 59 (49 - 96) | 0.24 |
| **τNa^+^_U_ (min)** | 120 (100 - 140) | 190 (180 - 190) | <0.0001 |
| **HR (bpm)** | 76 (71 - 89) | 86 (72 - 105) | 0.29 |
| **MAP (mmHg)** | 80 (72 - 93) | 89 (80 - 101) | 0.02 |
| **CVP (mmHg)^a^** | 7 (5 - 10) | 9 (6 - 12) | 0.02 |
| **GFR (ml/min)^a^** | 68 (62 - 85) | 63 (42 - 75) | 0.50 |
| **Baseline V_U_ (ml/kg)** | 0.8 (0.4 - 1.1) | 1.1 (0.6 - 1.6) | 0.10 |
| **V_U_ 1st hour (ml/kg/h)** | 4.9 (3.4 - 5.8) | 5.3 (4.3 - 7.4) | 0.40 |
| **V_U_ 2nd hour (ml/kg/h)** | 1.7 (1.2 - 2.5) | 2.4 (1.9 - 3.3) | 0.04 |
| **V_U_ 3rd hour (ml/kg/h)** | 0.9 (0.7 - 1.1) | 1.5 (1.0 - 1.9) | 0.02 |
| **Baseline FeNa^+^_U_ (%)^a^** | 0.4 (0.1 - 0.8) | 0.8 (0.3 - 2.0) | 0.05 |
| **FeNa^+^_U_ 1st hour (%)^a^** | 7.3 (4.8 - 9.2) | 8.8 (6.2 - 12.9) | 0.17 |
| **FeNa^+^_U_ 2nd hour (%)^a^** | 2.1 (1.1 - 2.8) | 4.3 (2.2 - 6.7) | 0.01 |
| **FeNa^+^_U_ 3rd hour (%)^a^** | 0.7 (0.3 - 1.0) | 1.9 (0.9 - 3.9) | 0.01 |
| **3 hours FeNa^+^_U_ (%)^a^** | 3.3 (2.3 - 4.8) | 4.7 (3.5 - 7.9) | 0.02 |
| **Total V_U_ (ml)** | 488 (409 - 614) | 573 (428 - 677) | 0.20 |
| **3 hours V_U_ (ml/kg)** | 2.3 (2.0 - 3.4) | 3.4 (2.5 - 4.0) | 0.10 |

Overall study population (*Single-Dose-Group*, n = 39) was divided according to the time constant of the decrease in urinary sodium concentration after furosemide administration. Data are reported as median value and interquartile ranges. ^a^ Data on CVP were available for 37 patients, whereas data on GRF and FeNa^+^ for 34 patients. Wilcoxon Signed Rank test was performed. *Abbreviations:* Na^+^_U_ = urinary sodium concentration; τNa^+^_U_ = time constant of urinary sodium concentration decrease; HR = heart rate; MAP = mean arterial pressure; CVP = central venous pressure; GFR = glomerular filtration rate; V_U_ = urinary output; FeNa^+^_U_ = fractional excretion of sodium.

**Supplementary Table S3 - Urinary electrolyte, pH and anion gap profile at baseline and during 8-hour furosemide administration**

|  | **Baseline** | **0-3**  **hours** | **3-5.5**  **hours** | **5.5-8**  **Hours** | **p** | **Median**  **8 hours** |
| --- | --- | --- | --- | --- | --- | --- |
| **[Na^+^]_U_ (mEq/L)** | 57 (24-102) | 133 (111-142)* | 73 (44-118) | 75 (28-121) | < 0.001 | 110 (93-137) |
| **[K^+^]_U_ (mEq/L)** | 63 (47-75) | 27 (20-38)* | 42 (36-51)* | 43 (36-49)* | < 0.001 | 32 (25-41) |
| **[Cl^-^]_U_ (mEq/L)** | 112 (64-131) | 133 (123-143)* | 111 (66-131) | 110 (53-124) | < 0.001 | 125 (112-132) |
| **[NH_4_^+^]_U_ (mEq/L)** | 8.3 (1.8-13.4) | 7.6 (6.1-11.2) | 9.1 (5.5-16.6) | 6.2 (4.4-13.4) | 0.31 | 8.0 (6-12) |
| **AG_U_ (mEq/L)** | 14 (-3-36) | 20 (7-27) | 14 (-3-25) | 22 (6-42) | 0.20 | 16 (6-30) |
| **Diuresis (ml/kg/h)** | 0.9 (0.4-1.3) | 2.6 (1.7-3.6)* | 0.9 (0.7-1.1) | 0.9 (0.7-1.2) | < 0.001 | 1.7 (1.2-2.3) |
| **Na^+^_U_ (µEq/Kg/min)** | 0.7 (0.1-1.2) | 5.9 (3.5-8.4)* | 1.2 (0.7-1.8) | 0.8 (0.4-2.2) | < 0.001 | 3.1 (2.0-5.1) |
| **K^+^_U_ (µEq/Kg/min)** | 0.8 (0.4-1) | 1.1 (0.9-1.5)* | 0.7 (0.5-0.8) | 0.6 (0.5-0.8) | < 0.001 | 0.9 (0.8-1.0) |
| **Cl^-^_U_ (µEq/Kg/min)** | 1.3 (0.6-2.1) | 6.5 (3.9-9.2)* | 1.8 (0.9-2.3) | 1.2 (0.6-2.5) | < 0.001 | 3.5 (2.5-5.2) |
| **NH_4_^+^_U_ (µEq/Kg/min)** | 0.05 (0.01-0.27) | 0.36 (0.22-0.48)* | 0.16 (0.08-0.28) | 0.12 (0.07-0.22) | < 0.001 | 0.26 (0.14-0.38) |

Data are reported as median value and interquartile ranges at baseline and of the average values during 30-min periods of intravenous administration of furosemide in the subgroup of patients in which urinary monitoring lasted 8 hours (*Long-Term-Group*, n = 24). Median 8 hours denotes median values of parameters recorded, as average, during the entire 8-hour period. One-way ANOVA for repeated measurements was performed. * P<0.05 vs. baseline. *Abbreviations:* Na^+^_U_ = urinary sodium; K^+^_U_ = urinary potassium; Cl^–^_U_ = urinary chloride; NH_4_^+^_U_ = urinary ammonium; AG_U_ = urinary anion gap; Fe = fractional excretion.

**Supplementary Table S4 – Urinary electrolyte, pH and anion gap profile at baseline and during multiple furosemide administration period.**

|  | **Basal** | **Average study** | **p** |
| --- | --- | --- | --- |
| **[Na^+^]_U_ (mEq/L)** | 73 (45 - 88) | 128 (118 - 140) | < 0.001 |
| **[K^+^]_U_ (mEq/L)** | 35 (27 - 49) | 24 (17 - 33) | < 0.001 |
| **[Cl ^-^ ]_U_ (mEq/L)** | 97 (84 - 115) | 129 (108 - 133) | 0.027 |
| **[NH_4_^+^]_U_ (mEq/L)** | 8.3 (4.9 - 12.2) | 7.3 (6.4 - 12.4) | 0.432 |
| **Diuresis (ml/kg/h)** | 1.0 (0.7 - 1.1) | 2.1 (1.6 - 2.5) | < 0.001 |
| **Diuresis (ml/h)** | 55 (45 - 66) | 121 (96 - 181) | < 0.001 |
| **AG_U (_mEq/L)** | 21 (15 - 35) | 26 (18 - 48) | 0.175 |
| **Na^+^_U_ (µEq/kg/min)** | 0.9 (0.5 - 1.5) | 3.7 (3.2 - 5.7) | < 0.001 |
| **K^+^_U_ ( µEq/kg/min )** | 0.6 (0.4 - 0.7) | 0.7 (0.7 - 1.0) | 0.042 |
| **Cl ^-^ _U_ ( µEq/kg/min )** | 1.6 (0.9 - 1.8) | 3.6 (2.8 - 5.3) | < 0.001 |
| **NH_4_^+^_U_ ( µEq/kg/min )** | 0.10 (0.08-0.19) | 0.23 (0.17-0.33) | 0.002 |
| **FeNa^+^_U_ (%)** | 0.4 (0.3 - 1.3) | 2.6 (1.9 - 4.4) | < 0.001 |
| **FeK^+^_U_ (%)** | 18 (12 - 22) | 22 (15 - 30) | 0.012 |
| **FeCl ^-^ _U_ (%)** | 1.4 (0.7 - 1.8) | 3.4 (2.8 - 4.4) | < 0.001 |
|  |  |  |  |

Data are reported as median value and interquartile ranges at baseline and of the average values recorded during the entire period of urinary monitoring for the subgroup of patients receiving multiple administrations (*Multiple-Dose-Group*, n = 11). Wilcoxon Signed Rank test was performed. *Abbreviations:* Na^+^_U_ = urinary sodium; K^+^_U_ = urinary potassium; Cl^–^_U_ = urinary chloride; NH_4_^+^_U_ = urinary ammonium; AG_U_ = urinary anion gap; Fe = fractional excretion.
